# Editorial: Physiological, Molecular and Genetic Perspectives of Chilling Tolerance in Horticultural Crops

**DOI:** 10.3389/fpls.2020.602144

**Published:** 2020-12-10

**Authors:** Isabel Lara, Maria F. Drincovich, Diane M. Beckles, Shifeng Cao

**Affiliations:** ^1^Postharvest Unit-AGROTÈCNIO, Universitat de Lleida, Lleida, Spain; ^2^Center for Photosynthetic and Biochemical Studies (CEFOBI, CONICET-Rosario National University), Rosario, Argentina; ^3^Department of Plant Sciences, MS3, University of California, Davis, Davis, CA, United States; ^4^College of Biological and Environmental Sciences, Zhejiang Wanli University, Ningbo, China

**Keywords:** cold stress, -omics, photosynthesis, reactive oxygen species, stress hormones, transcription factors, superficial scald

## Introduction

Horticultural crops have high economic, and enrich our lives through their aesthetic and nutritional value. Many horticultural species originate from tropical regions and are sensitive to cold at every stage of their lifecycle. Cold stress leads to lower productivity and post-harvest losses in these species, with poor economic and environmental outcomes.

Better understanding of the protective mechanisms mediated by hormonal and other signaling pathways (Akhtar et al., 2012) may offer solutions to reduce cold-stress induced losses. The papers included in this collection illustrate this concept, examining natural cold-tolerance mechanisms and practical ways for growers to alleviate chilling stress and to reduce crop losses. The studies were remarkably diverse in terms of the species studied (i.e., tomato, longan, tung tree, lowbush blueberry, and apple), plant organs examined (i.e., seedlings, leaf, and fruit), and approaches used (i.e., reverse genetics, the systems biology, physiology, and biochemistry).

The papers encompassed the use of (1) basic science, aimed at identifying key genes and their roles in cold signal transduction and protective pathways in fruit and photosynthetic tissues; (2) reverse genetics for proof-of-concept on the hypothesized role of a cold-tolerance transcription factor cloned from an understudied species; and (3) emerging technologies, by using exogenous hormones and signaling compounds to mitigate the harmful effects of chilling. These studies are described below.

## Cold Stress Tolerance Mechanisms

C-repeat binding factor (CBF) proteins constitute a transcription factor (TF) subfamily known to play a key role in plants against different types of abiotic stress including cold, heat, salinity or dehydration, and thus have been extensively studied. Overexpression of CBFs has been used for the development of genetically modified plants with enhanced stress tolerance and for the investigation of the molecular mechanisms underlying plant stress responses. Using this approach, Yang et al. found that overexpression of three newly identified longan (*Dimocarpus longan*) CBF genes (*DlCBF1, DlCBF2*, and *DlCBF3*) enhanced cold tolerance in *Arabidopsis* by increasing the content of the osmoprotectant proline, reducing the accumulation of reactive oxygen species (ROS), and stimulating the expression of cold-responsive genes. The fact that longan, a cold-sensitive species, showed low expression levels for these three genes, suggests a possible strategy for genetic improvement of cold tolerance in this crop.

Cold storage of apples is often used to extend post-harvest storage; however, it leads to superficial scald development, which is a major physiological disorder characterized by necrosis of the hypodermal cortical tissue. Karagiannis et al. applied a multiomics systems approach and created regulatory module networks to compare scald-affected and healthy apple phenotypes. Individual and combinatorial treatments with ozone (O_3_), which induced scald symptoms, and 1-methylcyclopropene (1-MCP), which reversed O_3_-stimulated scald effect, were used to identify pathways and gene-to-protein-to-metabolite networks involved in scald prevention and sensitivity. Importantly, 1-MCP-induced scald tolerance correlated with the expression of genes involved in photosynthesis, stress responses, flavonoid biosynthesis, and ethylene signaling in apple peel and key TFs that may control some of these processes. This study represents an important contribution for future functional studies to develop improved apple cultivars to superficial scald.

The acquisition of cold tolerance under conditions of varying light quality is essential for plants growing in regions with seasonal variation in both temperature and light (Yamori, 2016). Photoinhibition, i.e., the downregulation of the electron transport chain, reduces plant productivity, but safeguards the photosynthetic apparatus during cold and light stress (Tsonev et al., 2003; Guidi et al., 2019). Wang et al. investigated the role of light quality, specifically, low red to far-red ratios (L-R/FR), on photoprotection during cold stress in tomato. They showed that L-R/FR activated two pathways associated with cyclic electron flow (CEF): the PGR5/PGRL1A- and NDH-dependent complexes, respectively. These CEF complexes help to reduce cold-induced photodamage of the photosynthetic machinery by accelerating the thermal dissipation of excess energy, enhancing ROS scavenging, and reducing the hyper-reduction of the electron transport chain. This work therefore provides a better understanding of the mechanistic relationship between varying light quality and low temperature in plant photosynthetic performance in temperate climates when seasonal variation induces these conditions.

## Chilling Injury-Alleviating Technologies

Spring frosts cause important economic losses in many fruit-producing areas of the world, and there is interest in identifying feasible approaches to mitigate these risks. Ethylene controls fruit ripening in climacteric species (Johnson and Ecker, 1998) but it also plays an important role in plant stress responses (Wang et al., 2002; Harkey et al., 2019). Published literature on the use of ethylene or ethylene-based compounds for protecting temperate fruit orchards against frost damage was reviewed (Liu and Sherif). Experimental evidence of ethylene modulation of bud dormancy and blooming were presented and discussed. It was suggested that ethylene-delayed bloom and the associated frost protection may result from either (a) the slowing down of floral bud responsiveness to seasonal temperature changes, (b) an antagonistic interaction with other hormones such as abscisic acid or gibberellins, (c) plant sensing of exogenous ethylene as a stress signal leading to longer dormancy, or (d) ethylene-enhanced ROS accumulation resulting in extended bus dormancy.

Because chilling stress in plants often leads to ROS accumulation, the questions arises whether improving the antioxidant capacity of tissues by the exogenous application of antioxidant treatments may help improve tolerance to cold as well as to other types of abiotic stress. To this purpose, Tang et al. treated lowbush blueberry (*Vaccinium angustifolium*) seedlings with hydrogen sulfide (H_2_S), and found that treated plantlets performed better under low temperatures than the untreated controls, as shown by the alleviation of membrane peroxidation, the reduction of chlorophyll and carotenoid degradation, and the lessening of photosystem I and II photoinhibition. Conversely, the application of hypotaurine, a H_2_S scavenger, aggravated the oxidative symptoms under cold stress.

Brassinolide (BR) is an important plant stress hormone shown to promote plant resistance to low-temperature environments. Zhang et al. investigated the effects of exogenous BR on the photosynthetic characteristics, leaf anatomical structure, and chloroplast ultrastructure of two species of tung tree seedlings under different temperature conditions. The results suggested that long-term low temperatures significantly reduced the photosynthetic efficiency of tung tree seedlings, affecting the formation of the internal structure of plant leaves and destroying the integrity and function of the chloroplast. To prevent this, external application of BR to tung tree seedlings could enhance the photosynthetic potential of tung trees by maintaining the stability of the leaf structure and morphology and alleviating the damage caused by cold injury.

In summary, the papers in this collection illustrated the breadth of research aimed at understanding chilling responses in horticultural crops, but more importantly provided new insights that will further our future basic and applied research in this area.

## Author Contributions

All authors listed contributed directly to manuscript preparation, and revised and approved it before submission.

## Conflict of Interest

The authors declare that the research was conducted in the absence of any commercial or financial relationships that could be construed as a potential conflict of interest.

## References

[B1] Akhtar M. Jaiswal A. Taj G. Jaiswal J. P. Qureshi M. I. Singh N. K. (2012). DREB1/CBF transcription factors: their structure, function and role in abiotic stress tolerance in plants. J. Genet. 91, 385–395. 10.1007/s12041-012-0201-3 23271026

[B2] Guidi L. Piccolo E. L. Landi M. (2019). Chlorophyll fluorescence, photoinhibition and abiotic stress: does it make any difference the fact to be a C3 or C4 species? Front. Plant Sci. 10:174 10.3389/fpls.2019.00174 30838014 PMC6382737

[B3] Harkey A. F. Yoon G. M. Seo D. Y. DeLong A. Muday G. K. (2019). Light modulates ethylene synthesis, signaling, and downstream transcriptional networks to control plant development. Front. Plant Sci. 12:1094 10.3389/fpls.2019.01094 PMC6751313 31572414

[B4] Johnson P. R. Ecker J. R. (1998). The ethylene gas signal transduction pathway: a molecular perspective. Ann. Rev. Genet. 32, 227–254. 10.1146/annurev.genet.32.1.227 9928480

[B5] Tsonev T. Velikova V. Georgieva K. Hyde P. F. Jones H. G. (2003). Low temperature enhances photosynthetic down-regulation in French bean (*Phaseolus vulgaris* L.) plants. Ann. Bot. 91, 343–352. 10.1093/aob/mcg020 12547687 PMC4244959

[B6] Wang K. L.-C. Li H. Ecker J. R. (2002). Ethylene biosynthesis and signaling networks. Plant Cell 14, S131–S151. 10.1105/tpc.001768 12045274 PMC151252

[B7] Yamori W. (2016). Photosynthetic response to fluctuating environments and photoprotective strategies under abiotic stress. J. Plant Res. 129, 379–395. 10.1007/s10265-016-0816-1 27023791

